# Innate Activation of IFN-γ—iNOS Axis During Infection With *Salmonella* Represses the Ability of T Cells to Produce IL-2

**DOI:** 10.3389/fimmu.2020.00514

**Published:** 2020-03-25

**Authors:** Jitender Yadav, Neha Dikshit, Sana Ismaeel, Ayub Qadri

**Affiliations:** Hybridoma Laboratory, National Institute of Immunology, New Delhi, India

**Keywords:** T cell, *Salmonella* Typhimurium, IL-2, IFN-γ, iNOS, nitric oxide, splenocytes

## Abstract

Pathogenic *Salmonella* serovars are a major cause of enteric illness in humans and animals, and produce clinical manifestations ranging from localized gastroenteritis to systemic disease. T cells are a critical component of immunity against this intracellular pathogen. The mechanisms by which *Salmonella* modulates T-cell—mediated immune responses in order to establish systemic infection are not completely understood. We show that infection of mice with *Salmonella enterica* serovar Typhimurium (*S*. Typhimurium) suppresses IL-2 and increases IFN-γ and IL-17 production from T cells activated *in vivo* or *ex vivo* through the T cell receptor. Infection with *S*. Typhimurium brings about recruitment of CD11b^+^Gr1^+^ suppressor cells to the spleen. *Ex vivo* depletion of these cells restores the ability of activated T cells to produce IL-2 and brings secretion of IFN-γ and IL-17 from these cells back to basal levels. The reduction in IL-2 secretion is not seen in IFN-γ^−/−^ and iNOS^−/−^ mice infected with *Salmonella*. Our findings demonstrate that sustained innate activated IFN-γ production during progression of infection with *Salmonella* reduces IL-2—secreting capability of T cells through an iNOS-mediated signaling pathway that can adversely affect long term immunity against this pathogen.

## Introduction

Pathogenic *Salmonella* serovars produce different clinical manifestations depending upon *Salmonella* serovar and the type of host. *S*. Typhi infection in humans causes typhoid fever while infection with non-typhoidal *Salmonella* serovar, *S*. Typhimurium, produces only self-limiting gastroenteritis in humans. On the other hand, *S*. Typhi does not establish infection in mice while *S*. Typhimurium causes a systemic infection analogous to human typhoid. Therefore, this animal model has been extensively used to understand pathogenesis of human typhoid and to identify immune responses that are generated during infection with pathogenic *Salmonella* (1). Infection of mice with *S*. Typhimurium elicits a potent innate immune response resulting in secretion of cytokines and chemokines and generation of antimicrobial molecules (2). This innate response is produced following activation of membrane-bound TLRs including TLR4 and TLR5, which recognize LPS and flagellin respectively, and intracellular cytosolic sensors like NLRC4, NLRP3, and Caspase-11 (3, 4). NLR activation brings about activation of caspase-1 that triggers inflammatory cell death called pyroptosis accompanied by release of IL-1β and IL-18 (5). The responses activated through these innate sensors are also essential for generation and maintenance of the adaptive immune responses which provide long term immunity against this pathogen (6). Mice lacking αβ T cells, MHC class-II or T-bet^+^ Th1 cells are incapable of clearing attenuated *Salmonella* and develop chronic disease (7–9). In contrast, mice which lack γδ T cells, B cells or MHC class-I can resolve primary infection with attenuated *Salmonella* (10, 11). *Salmonella*-specific CD4 and CD8 T cells are generated during infection with attenuated bacteria in mice (12–14). Adoptive transfer experiments have shown that CD4 T cells are more important than CD8 T cells in clearing *Salmonella* infection. These cells mediate immunity through cytokines IL-17 and IFN-γ or *via* direct killing of *Salmonella*-infected cells (15, 16). CD4 T cells are also the chief source of IFN-γ that is produced in an antigen independent, IL-18—dependent manner during infection with *Salmonella* (17–19). Mice lacking IFN-γ have higher bacterial burden than wild type mice (20).

*Salmonella* has evolved many strategies to counter or evade immune responses during establishment of infection. However, while the modulation of innate immune responses during infection with this pathogen is well studied (21–24), the strategies which *Salmonella* employs to evade T-cell—mediated immune responses have not been investigated in detail (25–27). *Salmonella* has been shown to inhibit CD4 T cell activation *in vitro* by bringing about downregulation of the T cell receptor and by inducing negative modulators of T cell activation (28–30). It has also been shown to inhibit antigen processing and presentation from dendritic cells *in vitro* (31). The effectors of *Salmonella* pathogenicity island - 2 (SPI-2) have been reported to suppress migration of dendritic cells to the site of infection, downregulate CD4 and CD8 T cell responses and polarize macrophage functions (23, 24, 32, 33). However, the exact mechanism by which this pathogen modulates T cell responses during *in vivo* infection and the interplay of T cells with other immune cells particularly mononuclear phagocytes which host *Salmonella* during *in vivo* infection remain poorly understood. We show here that sustained innate immune activation of IFN-γ during establishment of infection with *S*. Typhimurium in mice specifically reduces the ability of T cells to secrete IL-2 upon activation through the TCR.

## Materials and Methods

### Mice and Bacterial Strains

Wild type C57BL/6 and MyD88^−/−^, iNOS^−/−^, IFN-γ^−/−^ strains (on C57BL/6 background) were procured from the Jackson laboratory, USA and maintained at the Small Animal Facility of the National Institute of Immunology. The experiments with mice were carried out according to the guidelines provided by the Institutional Animal Ethics Committee (IAEC). *Salmonella* Typhimurium SL1344 strain was provided by Prof. Emmanuelle Charpentier, Department of Microbiology and Genetics, University of Vienna, Austria (now at the Max Planck Institute for Infection Biology in Berlin). GFP-expressing SL1344 was provided by Dr. Amitabha Mukhopadhyay, Cell Biology Laboratory, National Institute of Immunology, New Delhi, India. Bacteria were cultured in LB medium supplemented with streptomycin at 37°C with shaking at 220 rpm for 12–14 h. Bacterial loads were determined by plating tissue lysates on *Salmonella* – *Shigella* (SS) agar plates.

### Preparation of *Salmonella* Sonicates

Bacteria grown in LB medium were pelleted by centrifuging at 8000 × g for 5 min. The pellet was washed 3 times with PBS, resuspended in ice cold PBS and sonicated on ice with brief pulses of sonication and cooling, 1 min each. This cycle was repeated 5 times. The sonicate was centrifuged at 10,000 × g for 20 min at 4°C. The supernatant was filtered through 0.22 μ membrane and protein concentration was determined by BCA kit from Pierce, according to the manufacturer's instructions.

### *In vivo* Infection With *S*. Typhimurium, and Analysis of T Cell Activation

Six to eight week old C57BL/6 mice were infected intraperitoneally or orally with 100 CFU or 10^8^ CFU of *S*. Typhimurium respectively. Spleens excised from uninfected and infected mice were placed in a petri-dish containing RPMI and crushed using the plunger of a syringe. Single cell suspension was prepared by passing 2–3 times through a 21-gauge needle. Cells were pelleted at 800 × g for 5 min, washed twice with RPMI and resuspended in RPMI-10. Splenocytes from uninfected and infected mice were plated in triplicate at a density of 0.3 × 10^6^ cells per well in a 96-well cell culture plate. Anti-CD3 antibody (145-2C11 obtained from the ATCC, and antibody purified from the culture supernatant using Protein G—Sepharose affinity column) was added to the splenocytes and cells were cultured in RPMI-10 supplemented with gentamycin (100 μg/ml). Culture supernatants were collected 32 h post-stimulation and analyzed for IL-2, IFN-γ, and IL-17 by ELISA. In some experiments, T-cell stimulation was carried out after depleting CD11b^+^Gr1^+^ cells.

### Determination of Nitric Oxide (NO) Levels Using Griess Reagent

Culture supernatants from uninfected and *S*. Typhimurium-infected splenocytes were collected at 32 h. Fifty microliters of each sample was mixed with 50 μl of Griess reagent and the optical density was measured at 560 nm. NaNO_2_ was used as the standard.

### Flow Cytometric Analysis of Cell Populations in the Spleen

Macrophages, B cells, and T cell populations in the spleens of uninfected and *Salmonella*-infected mice were analyzed by flow cytometry using cell type specific antibodies. Briefly, splenocytes were adjusted to 1 × 10^6^ cells and stained with specific antibodies against cell surface markers CD11b (M1/70 clone), Gr1 (RB6-8C5 clone), CD4 (GK1.5 clone), CD8 (53-6.7 clone) and B220 (RA3-6B2 clone), or isotype matched antibodies for 60 min at 4°C. After washing, ten thousand cells were enumerated in a flow cytometer (FACS Verse or Accuri C6; BD Pharminogen, USA). Compensation in two color flow cytometry was done using cells labeled with single fluorophore.

### Cell Proliferation

To investigate effect of *Salmonella* infection on T cell proliferation, splenocytes from uninfected and *Salmonella*-infected mice were plated in triplicate at a density of 0.3 x 10^6^ cells/well in a 96-well plate and stimulated with anti-CD3 antibody. Cells were incubated for 32 h at 37°C, and pulsed with ^3^H-thymidine (0.5 μCi/well). After 12–15 h at 37°C, the amount of radioactivity incorporated in cells was determined by liquid scintillation spectroscopy (Perkin Elmer, Waltham, MA, USA).

### Depletion of Gr1^+^ Cells From *S*. Typhimurium-Infected Mouse Splenocytes

Gr1^+^ cells were depleted from splenocytes by positive selection using MACS cell separation kit (Miltenyi Biotec, Auburn, CA). Briefly, splenocytes from *S*. Typhimurium—infected mice were incubated with biotin conjugated anti-Gr1 antibody. Cells were washed with PBS, and incubated with avidin magnetic beads for 30 min at 4°C. After washing, cell suspension was passed through a MACS LD column and flow through containing Gr1-depleted fraction was collected. The efficiency of depletion was checked by staining cells with PE-labeled anti-mouse Gr1 antibody and analyzed in a flow cytometer (BD-Accuri C6).

### *In vivo* Administration of Anti-CD3 Antibody

Mice were infected intraperitoneally with 100 CFU of *S*. Typhimurium, and anti-CD3 antibody or an isotype control antibody (obtained from BioLegend, USA; 10 μg/mouse) was administered intraperitoneally in uninfected and infected mice (on day 5 of *Salmonella* infection). Sera were collected after 2 h of antibody administration and analyzed for IL-2 and IFN-γ by ELISA.

### Statistical Analysis

Student's *t*-test with a two tailed distribution and type 3 test for unequal variances was used to calculate *p*-values. A *p*-value of < 0.05 was considered statistically significant. Data are expressed as mean ± SD. Error bars represent standard deviation (SD).

## Results

### Infection of Mice With *Salmonella* Typhimurium Differentially Modulates Secretion of IL-2 and Effector Cytokines From T Cells

**Figure 1:**
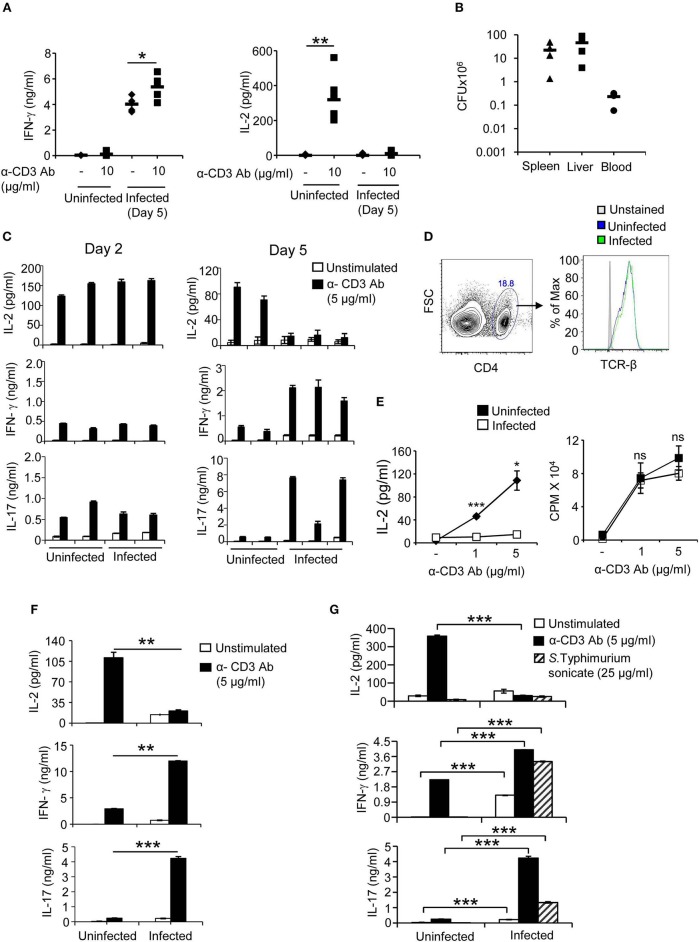
T cells from *S*. Typhimurium—infected mice secrete reduced amounts of IL-2, show normal proliferation and produce increased IFN-γ and IL-17 upon stimulation through the TCR. **(A)** C57BL/6 mice were infected intraperitoneally with 100 CFU of *S*. Typhimurium. On day 5, mice were injected with anti-CD3 antibody (10 μg/mouse). Sera were collected prior to and 2 h after antibody treatment and levels of IL-2 and IFN-γ were determined by ELISA. **(B)** Mice were infected intraperitoneally with 100 CFU of *S*. Typhimurium and bacterial load in spleen, liver and blood was determined on day 5 post infection by plating tissue lysates on SS agar plates. Numbers shown are bacteria per tissue for liver and spleen, and per ml for blood. **(C)** Mice were infected intraperitoneally with 100 CFU of *S*. Typhimurium. Splenocytes isolated on days 2 and 5 from uninfected and infected mice were stimulated with anti-CD3 antibody (5 μg/ml) *ex vivo*. Culture supernatants were collected after 32 h and levels of IL-2, IFN-γ, and IL-17 were determined by ELISA. Each bar (mean ± SD) represents a mouse, and data is representative of 3 independent experiments. **(D)** Splenocytes from uninfected and *S*. Typhimurium-infected (5 days) mice were incubated with PE-labeled anti-TCRβ and perCP-cy5.5-labeled anti-CD4 antibodies or PE-labeled isotype matched antibodies. After washing, cells were analyzed in a flow cytometer (BD, FACS Verse). The histograms show TCR-β expression on T cells gated for CD4. Data is representative of 3 mice per group from 3 independent experiments. **(E)** Splenocytes isolated from uninfected and *S*. Typhimurium *-* infected mice were stimulated with anti-CD3 antibody (5 μg/ml). Thirty-two hours later, cell-free supernatant was collected and analyzed for IL-2 by ELISA. Cells were pulsed with ^3^H-thymidine and the amount of radioactivity incorporated was analyzed after 16 h by liquid scintillation spectroscopy. **(F)** Mice were infected orally with 10^8^ CFU of *S*. Typhimurium and on day 6, splenocytes were stimulated with anti-CD3 antibody (5 μg/ml) *ex vivo*. 32 h later, culture supernatants were analyzed for IL-2, IFN-γ, and IL-17 by ELISA. **(G)** Splenocytes obtained on day 5 of *S*. Typhimurium infection were stimulated *ex vivo* with anti-CD3 antibody or bacterial sonicate prepared from *S*. Typhimurium. Culture supernatants were analyzed for IL-2, IL-17, and IFN-γ by ELISA. Data shown as mean ± SD in **(A)** is representative of 5 mice per group from 2 independent experiments, in **(B)** representative of 4 mice per group from 2 independent experiments and in **(E,F,G)** are representative of 3 mice per group from 3 independent experiments, ns, not significant. *p < 0.05, **p < 0.01, ***p < 0.005.

T cells and T cell-derived cytokines play a fundamental role in immunity against microbial pathogens including *Salmonella*. Many pathogens have therefore evolved strategies to evade immune responses produced by T cells. To study possible effects of *Salmonella* on T cell activation during systemic phase of infection in which this pathogen is largely present in spleen and liver, C57BL/6 mice were infected intraperitoneally with *S*. Typhimurium and on day 5, mice were injected with anti-CD3 antibody and cytokines IL-2 and IFN-γ were determined in sera. Sera obtained from mice infected with *S*. Typhimurium and injected with anti-CD3 antibody showed higher levels of IFN-γ as compared to sera from uninfected anti-CD3 antibody—treated mice (Figure 1A). In contrast, however, sera from infected mice treated with anti-CD3 antibody showed negligible levels of IL-2 as compared to sera from uninfected mice treated with anti-CD3 antibody suggesting that infection with *S*. Typhimurium might specifically suppress the ability of T cells to secrete IL-2 upon stimulation through the TCR (Figure 1A). Administration of an isotype matched control antibody did not result in any IL-2 secretion. As is normally seen during oral infection, infection through the intraperitoneal route also resulted in systemic dissemination of *Salmonella* (Figure 1B). The selective reduction in IL-2 was readily recapitulated with splenic T cells from mice infected with *S*. Typhimurium intraperitoneally and activated with anti-TCR antibody *ex vivo* (Figure 1C). Splenocytes obtained from mice on day 2 post infection produced IL-2, IFN-γ, and IL-17, levels of which were comparable with those produced by splenocytes from uninfected mice (Figure 1C). On the other hand, splenocytes isolated from mice after 5 days of infection with *S*.Typhimurium showed considerably reduced IL-2 secretion when compared with activated uninfected splenocytes (Figure 1C). Similar results were obtained in the presence of anti-CD28 antibody (data not shown). In contrast to IL-2, IFN-γ, and IL-17 were produced at higher levels by splenic cells from infected mice when compared with splenocytes from uninfected mice (Figure 1C). The levels of surface TCR in splenic T cells from uninfected and infected mice were comparable (Figure 1D), ruling out the possibility that impaired IL-2 secretion might be an outcome of reduced TCR expression in T cells from infected mice. Also and very significantly, there was no difference in the ability of splenocytes from uninfected mice and *S*. Typhimurium-infected mice to undergo proliferation upon activation through the TCR (Figure 1E). The reduction in IL-2 secretion and increase in IFN-γ production was also observed with splenocytes isolated from mice infected with *S*. Typhimurium orally (Figure 1F). These data established differential effects of *Salmonella* infection on IL-2 and effector cytokines. This dichotomy was also seen when splenocytes from mice infected with *S*. Typhimurium were stimulated with antigenic extract (sonicate) prepared from this pathogen. Splenocytes from infected mice did not produce any detectable IL-2 upon stimulation with *S*. Typhimurium sonicate *ex vivo* (Figure 1G). However, these cells produced small amounts of IFN-γ and IL-17 constitutively, which were enhanced several fold upon stimulation with *S*. Typhimurium sonicate (Figure 1G). These results demonstrated that infection with *S*. Typhimurium was specifically targeting production of IL-2 from T cells.

### CD11b^+^Gr1^+^ Cells Modulate Cytokine Secretion From T Cells

**Figure 2:**
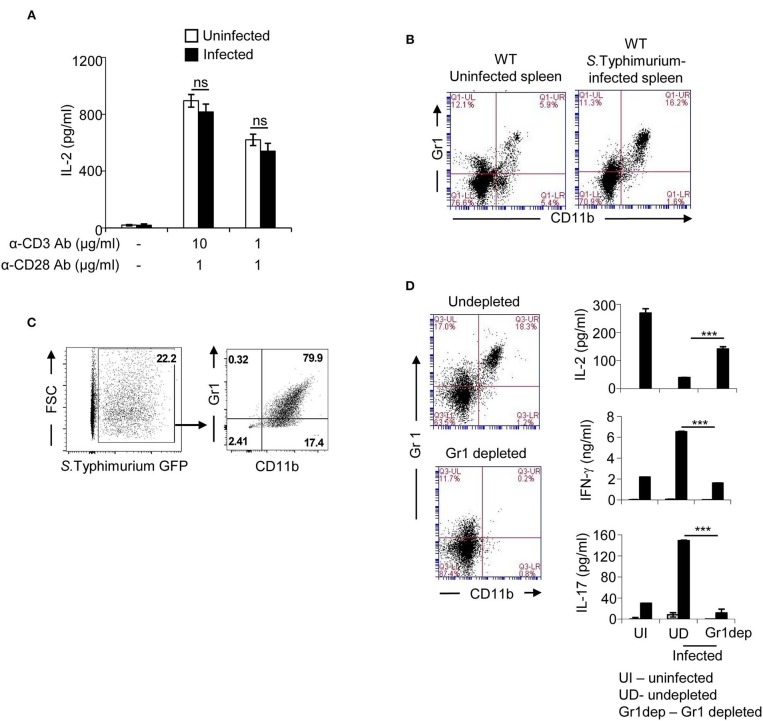
CD11b^+^Gr1^+^ splenic cells from *S*. Typhimurium—infected mice reduce IL-2 secretion from T cells. **(A)** T cells purified from spleens of uninfected and infected mice (day 5 of infection) were stimulated with plate coated anti-CD3 antibody and anti-CD28 antibody for 36 h. Culture supernatants were analyzed for IL-2 by ELISA. **(B)** Mice were infected intraperitoneally with 100 CFU of *S*. Typhimurium. Splenocytes isolated from uninfected or *S*. Typhimurium–infected mice were stained with FITC-labeled anti-CD11b and PE-labeled anti-Gr1 antibody, and analyzed by flow cytometry (BD Accuri C6). Data shown is representative of 3 mice per group from 4 independent experiments. **(C)** Mice were infected intraperitoneally with 100 CFU of GFP expressing *S*. Typhimurium. Splenocytes were stained with APC cy7-labeled anti-CD11b and PE-labeled anti-Gr1 antibody, and analyzed by flow cytometry (BD FACS Verse). Proportion of CD11b^+^Gr1^+^ cells containing GFP expressing *S*. Typhimurium is shown. Data shown is representative of 3 mice per group from 3 independent experiments. **(D)** Splenocytes from *S*. Typhimurium - infected mice were incubated with biotinylated anti-Gr1 antibody followed by Avidin-beads as per instructions provided by the manufacturer (Miltenyi Biotec). Undepleted splenocytes and Gr1-depleted splenocytes were stained with PE-labeled anti-Gr1 and FITC-labeled anti-CD11b antibodies, and analyzed by flow cytometry (BD Accuri C6). Cells were stimulated with anti-CD3 antibody and after 32 h, culture supernatants were collected, and levels of IL-2, IFN-γ, and IL-17 were analyzed by ELISA. Data shown as mean ± SD in **(A)** is representative of 3 mice per group from 3 independent experiments, and in **(D)** representative of 3 mice per group from 2 independent experiments. ns, not significant. ***p < 0.005.

To understand the mechanism responsible for reduced IL-2 secretion, purified T cells were activated with anti-CD3 antibody together with anti-CD28 antibody. T cells from uninfected and *Salmonella*—infected mice did not show any significant difference in IL-2 secretion upon stimulation through the TCR (Figure 2A). These results indicated that infection with *S*. Typhimurium did not affect the intrinsic ability of T cells to produce IL-2, nor did it indicate that T cells from infected mice were in a highly activated state capable of using up IL-2 and thereby resulting in reduced detection of this cytokine in the supernatant. Instead, these findings raised the possibility that one or more cell types of non-T cell origin present in the spleens of infected mice might be responsible for reducing IL-2 secretion from activated T cells. Flow cytometric analysis of splenocytes from mice infected with *S*. Typhimurium showed increased numbers of CD11b^+^Gr1^+^ positive cells (Figure 2B; Supplementary Figure 1). A large fraction of these cells harbored *Salmonella* (Figure 2C). These cells, collectively called myeloid—derived suppressor cells (MDSCs), are known to be immunosuppressive (34). We therefore analyzed the role of these cells in modulating cytokine secretion from T cells during infection with *Salmonella*. *Ex vivo* depletion of CD11b^+^Gr1^+^ cells from splenocytes obtained from *Salmonella*—infected mice resulted in considerable recovery in the ability of splenic T cells to secrete IL-2 upon stimulation through the TCR (Figure 2D). This depletion also resulted in reduction of IFN-γ and IL-17 from these T cells to levels similar to those produced by uninfected splenocytes (Figure 2D). These data revealed an immunoregulatory role for CD11b^+^Gr1^+^ cells in differentially modulating IL-2 and effector cytokines from splenic T-cells during infection with *Salmonella*.

### CD11b^+^Gr1^+^ Cells Down-Regulate Secretion of IL-2 From Splenic T Cells Through IFN-γ—iNOS Axis

**Figure 3:**
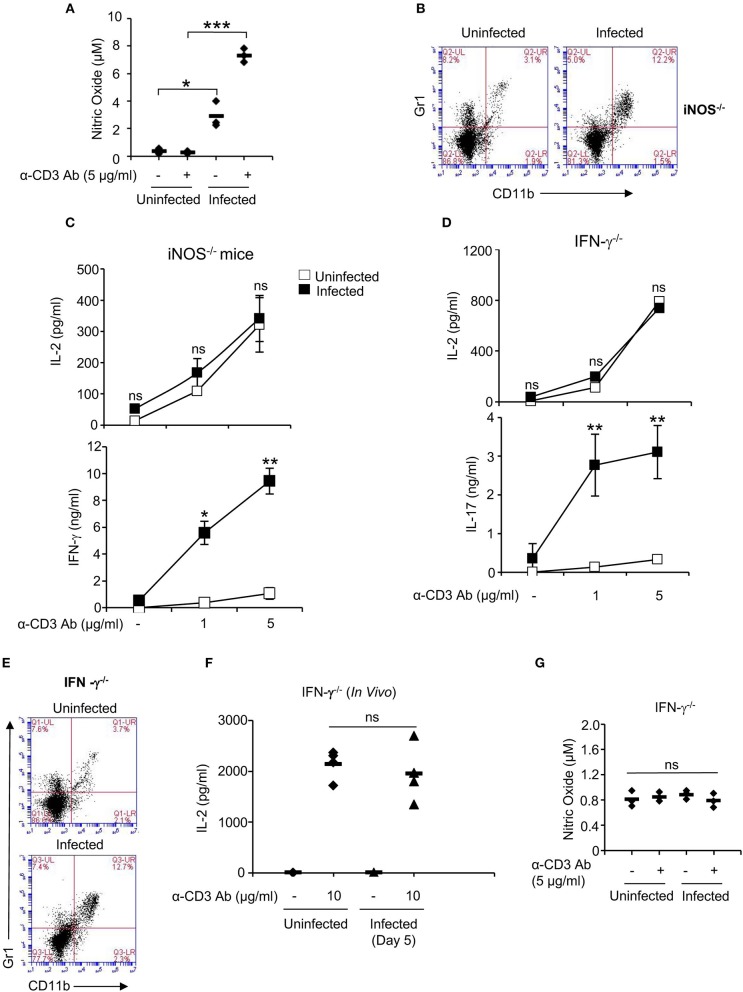
IFN-γ—NO axis suppresses the ability of activated T cells to secrete IL-2. **(A)** Splenocytes from uninfected and *S*. Typhimurium-infected mice were stimulated with anti-CD3 antibody *ex vivo* and after 32 h, NO levels were analyzed in the culture supernatants by Griess Reagant. **(B)** iNOS^−/−^ mice were infected intraperitoneally with 100 CFU of *S*. Typhimurium. Splenocytes isolated from uninfected or *S*. Typhimurium - infected mice were stained with FITC-labeled anti-CD11b and PE-labeled anti-Gr1 antibody and analyzed by flow cytometry (BD Accuri C6). **(C,D)** IFN-γ^−/−^ and iNOS^−/−^ mice were infected intraperitoneally with 100 CFU of *S*. Typhimurium. Splenocytes from uninfected and infected mice were stimulated with anti-CD3 antibody (1 μg and 5 μg/ml) *ex vivo*. Culture supernatants were analyzed for IL-2 and IFN-γ by ELISA. **(E)** Splenocytes from uninfected or *S*. Typhimurium–infected IFN-γ^−/−^ mice were stained with FITC-labeled anti-CD11b and PE-labeled anti-Gr1 antibody and analyzed by flow cytometry (BD Accuri C6). (**F**) Anti-CD3 antibody (10 μg/mouse) was injected intraperitoneally into IFN-γ^−/−^ uninfected and *S*. Typhimurium—infected mice. Sera collected prior to and 2 h post α-CD3 treatment were analyzed for IL-2 by ELISA. **(G)** Splenocytes from IFN-γ^−/−^ uninfected and *S*. Typhimurium-infected mice were stimulated with anti-CD3 antibody *ex vivo* and after 32 h, NO levels were analyzed in the culture supernatants. Data shown as mean ± SD in **(A,C,D,G)** is representative of 3 mice per group from 3 independent experiments, and in **(F)** representative of 4 mice per group from 2 independent experiments. Flow cytometry data in **(B,E)** is representative of 3 mice per group from 3 independent experiments. ns, not significant. *p < 0.05, **p < 0.01, ***p < 0.005.

CD11b^+^Gr1^+^ cells have been shown to exert their suppressive effect on T cells through production of various inhibitory molecules including arginase-1, inducible nitric oxide synthase (iNOS) and reactive oxygen species [ROS; (35)]. Splenocytes obtained from *Salmonella*-infected mice showed increased levels of NO, which increased further upon stimulation of cells with anti-CD3 antibody (Figure 3A). Therefore, we investigated role of NO in modulating IL-2 secretion from splenic T cells by infecting iNOS deficient with *S*. Typhimurium. Like WT mice, iNOS deficient mice also showed recruitment of CD11b^+^Gr1^+^ cells in the spleen and like WT mice, splenocytes from *S*. Typhimurium-infected iNOS-deficient mice showed increased IFN-γ secretion upon stimulation through the TCR (Figures 3B,C; Supplementary Figure 1). However, unlike WT mice, infection with *Salmonella* did not reduce the ability of activated T cells from these mice to produce IL-2 (Figure 3C), suggesting that NO produced by infected splenic CD11b^+^Gr1^+^ cells were responsible for downregulating IL-2 production from activated T cells.

**Figure 4:**
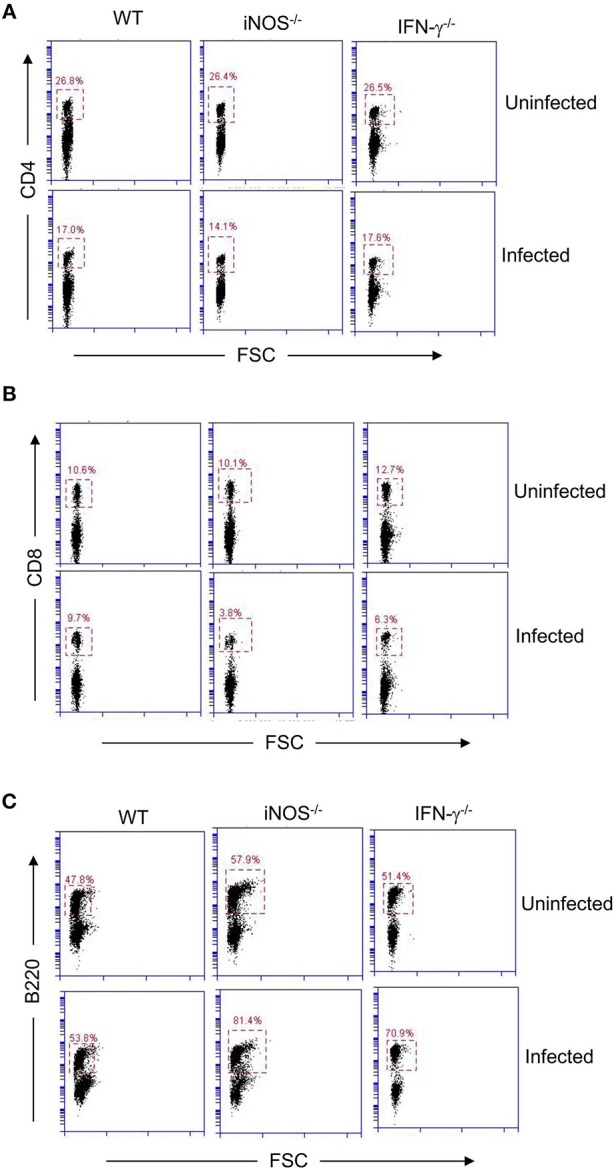
IFN-γ and iNOS regulate numbers of CD8 T cells and B220^+^ B cells during infection of mice with *S*. Typhimurium. C57BL/6 (WT), iNOS^−/−^ and IFN-γ^−/−^ mice were infected intraperitoneally with 100 CFU of *S*. Typhimurium. Splenocytes from uninfected and *S*. Typhimurium - infected mice were stained for **(A)** CD4 (with FITC-labeled anti-CD4 antibody) **(B)** CD8 (with PE-labeled anti-CD8 antibody) and **(C)** B220 (with FITC-labeled anti-B220 antibody). Cells were washed and analyzed by flow cytometry (BD Accuri C6). Data is representative of 3 mice per group from 2 independent experiments.

It is known that the release of NO from MDSCs is activated by IFN-γ (36). Therefore, we tested T cells from IFN-γ deficient mice infected with *S*. Typhimurium for their ability to produce IL-2. Similar to iNOS-deficient mice, splenocytes from *S*. Typhimurium—infected IFN-γ–deficient mice did not show impaired IL-2 secretion upon TCR activation even though CD11b^+^Gr1^+^ cells were present in the spleens of these mice (Figures 3D,E; Supplementary Figure 1). Like WT mice, activated T cells from these infected mice produced increased amounts of IL-17 (Figure 3D). These results suggested that *S*. Typhimurium exploits IFN-γ—iNOS axis to downmodulate IL-2 secretion from splenic T cells. To confirm the role for the IFN-γ—iNOS axis in downmodulating IL-2 production *in vivo*, serum levels of IL-2 were measured in *S*. Typhimurium—infected IFN-γ–deficient mice post intraperitoneal administration of anti-CD3 antibody. Consistent with the results obtained *ex vivo, S*. Typhimurium—infected IFN-γ–deficient mice did not show reduced IL-2 in serum (Figure 3F). IFN-γ mediated downregulation of IL-2 was almost exclusively through NO as T cells from iNOS^−/−^ mice produced high amounts of IFN-γ *ex vivo*, yet these cells were not defective in IL-2 secretion (Figure 3C). Likewise, IFN-γ deficient mice did not produce NO upon infection with *S*. Typhimurium (Figure 3G). In addition to recruitment of CD11b^+^Gr1^+^ cells, other changes in cellularity observed in WT mice infected with *S*. Typhimurium were also seen in IFN-γ^−/−^ and iNOS^−/−^ mice (Figures 3B,E, 4). Interestingly, while all three strains of mice showed reduced CD4 T cell numbers following infection with *S*. Typhimurium, infected iNOS^−/−^ and IFN-γ^−/−^ mice also had reduction in CD8 T cell numbers (Figure 4; Supplementary Figures 2, 3). The proportion of B220^+^ cells was higher in the knock-out strains of infected mice, however, the total number of B220^+^ cells per spleen was not different (Figure 4; Supplementary Figures 2, 3). These mice are known to be more susceptible to infection with *Salmonella* and show increased tissue bacterial burden (37–39).

## Discussion

Our results show that infection of mice with *S*. Typhimurium renders T cells specifically incapable of producing IL-2. This incapability is seen not only upon stimulation of cells with *Salmonella* antigens but also upon activation with anti-TCR antibody suggesting that the effect of infection on IL-2—producing ability is not restricted to antigen—specific T cells. This effect is not due to imprinting of an intrinsic defect in T cells from infected mice but it is brought about through interaction of these cells with CD11b^+^Gr1^+^ MDSCs which are recruited to the spleen during infection with *S*. Typhimurium. Significantly, these cells also impart splenic T cells an increased ability to produce IFN-γ and IL-17 upon activation through the TCR. The latter effect is produced primarily because MDSCs from infected mice serve as a source of inflammatory cytokines like IL-6, IL-18, IL-12, IL-23, & IL-1β, which can act on T cells and bring about secretion of IFN-γ and IL-17 (34, 40). Therefore, these MDSCs might also contribute to bacterial clearance albeit to a small degree. MDSCs have been shown to suppress T-cell responses in several infection and non-infection models of inflammation (41, 42), and their suppressive activity has been assigned to various factors including nitric oxide (NO), arginase-1, reactive oxygen species and peroxynitrite (34). Several studies have previously shown that NO produced by *Salmonella*-infected cells can suppress T-cell responses but those studies were carried out using mostly *in vitro* models (43, 44). MDSCs are now recognized as a heterogenous population of myeloid origin with subpopulations of monocytic and granulocytic phenotype. These subpopulations have been shown to differentially modulate T-cell functions in different disease models (34). Tam et al. (44) reported immunosuppressive properties with CD11b^+^Gr1^+^ cells recruited to the spleen in a persistent model of *S*. Typhimurium infection. This inhibitory capability was found to be associated with CD11b^+^Ly6C^hi^Ly6G^−^ fraction which had mononuclear morphology and produced NO. On the other hand, CD11b^+^Ly6C^int^Ly6G^+^ fraction of CD11b^+^Gr1^+^ cells with polymorphonuclear morphology did not suppress T cell activation (44). In tumor models, a large proportion of MDSCs have granulocytic morphology and show increased expression of arginase and iNOS (34, 45). In future studies, it will be interesting to see if the functional heterogeneity reported by Tam et al. (44). in the persistent model is also observed in the acute model of *Salmonella* infection used in the present study. Irrespective of that possible heterogeneity, our findings not only establish the role of iNOS in downregulating the ability of T cells to secrete IL-2 *in vivo* but also assign a critical role to an otherwise key antibacterial cytokine IFN-γ in this downregulation. Unlike previous reports, we did not see any defect in T cell proliferation in presence of CD11b^+^Gr1^+^ cells. This could be due to reduced levels of NO produced by these cells in our model of infection; higher levels of NO have been implicated in suppressing T cell proliferation (46). Alternatively, other cytokines produced by these cells might neutralize the ability of NO to suppress T cell proliferation. The second possibility looks more plausible considering that the number of splenic T cells in infected mice was significantly reduced yet these cells proliferated as well as T cells from uninfected mice following *ex vivo* stimulation with anti-TCR antibody. This phenomenon was evident *in vivo* as well. Mice deficient in IFN-γ and iNOS infected with *S*. Typhimurium had considerably reduced splenic T cell numbers yet their IL-2 response was comparable with that of uninfected mice pointing once again to a negative regulatory role for NO in IL-2 secretion, and reiterating that cytokines produced by CD11b^+^Gr1^+^ cells might increase IL-2 secretion from T cells in the absence of NO. The exact mechanism by which NO selectively downregulates IL-2 secretion is not clear. A recent study has suggested that CD11b^+^Gr1^+^ MDSCs produced during cancers might inhibit IL-2 production through nitration of lck (45).

IL-2 is known to play a key role in several critical T-cell functions. It promotes proliferation as well as survival of T cells and contributes to generation of memory T cells, provides help for CD8 T cell activation and their differentiation into effector cells and regulates generation and homeostasis of regulatory T cells (47–49). It also stimulates proliferation and activation of NK cells and together with IL-12 enhances their cytotoxic activity (50–52). Recently, it has been shown that IL-2 may be preferentially secreted by T cells which go on to become Tfh cells and this IL-2 is critical for differentiation of T cells into Th1 type of effector T cells (53). Therefore, selective downregulation of the ability of T cells by MDSCs to secrete IL-2 upon activation would have significant implications for long term immunity against infection with *Salmonella*. Heithoff et al. have shown that reduced expansion of MDSCs correlates with better protective immunity with a *Salmonella* vaccine (54). Significantly, IFN-γ, which is a crucial antibacterial cytokine, seems to be at the forefront of reducing IL-2—producing capability of T cells. Mice deficient in IFN-γ and iNOS are more susceptible to infection with *Salmonella* (55, 56). However, IFN-γ levels increase progressively during infection with *S*. Typhimurium and the levels correlate with higher bacteremia. Our findings suggest that sustained IFN-γ produced through innate immune activation during infection with *Salmonella* (may be other pathogens as well) might be a significant contributory factor to dampening of long term immunity against this pathogen. Persistent model of *Salmonella* infection would provide a time window to investigate in future if sustained innate immune response might come at the cost of diminished adaptive immunity. Significantly, since reduced IL-2—secreting capability of T cells is seen independent of antigen specificity; this modulation might also affect immune responses against other pathogens which a host might encounter during infection with *Salmonella*.

## Data Availability Statement

All datasets generated for this study are included in the article/Supplementary Material.

## Ethics Statement

The animal study was reviewed and approved by the Institutional Animal Ethics Committee of the National Institute of Immunology.

## Author Contributions

AQ conceived and supervised the study. JY, ND, and AQ designed experiments. JY, ND, and SI performed experiments and prepared data for publication. JY, ND, SI, and AQ analyzed the data and wrote the manuscript. All authors approved the manuscript.

### Conflict of Interest

The authors declare that the research was conducted in the absence of any commercial or financial relationships that could be construed as a potential conflict of interest.

## Abbreviations

IL: Interleukin
IFN-γ: Interferon gamma
MDSCs: myeloid-derived suppressor cells
iNOS: inducible nitric oxide synthase
TLR: Toll like receptor.
