# Supplementary figures and images for: Innate Activation of IFN-γ—iNOS Axis During Infection With Salmonella Represses the Ability of T Cells to Produce IL-2
Source: Front Immunol. 2020 Mar 25;11:514. doi: 10.3389/fimmu.2020.00514 (PMC7109407; doi:10.3389/fimmu.2020.00514)

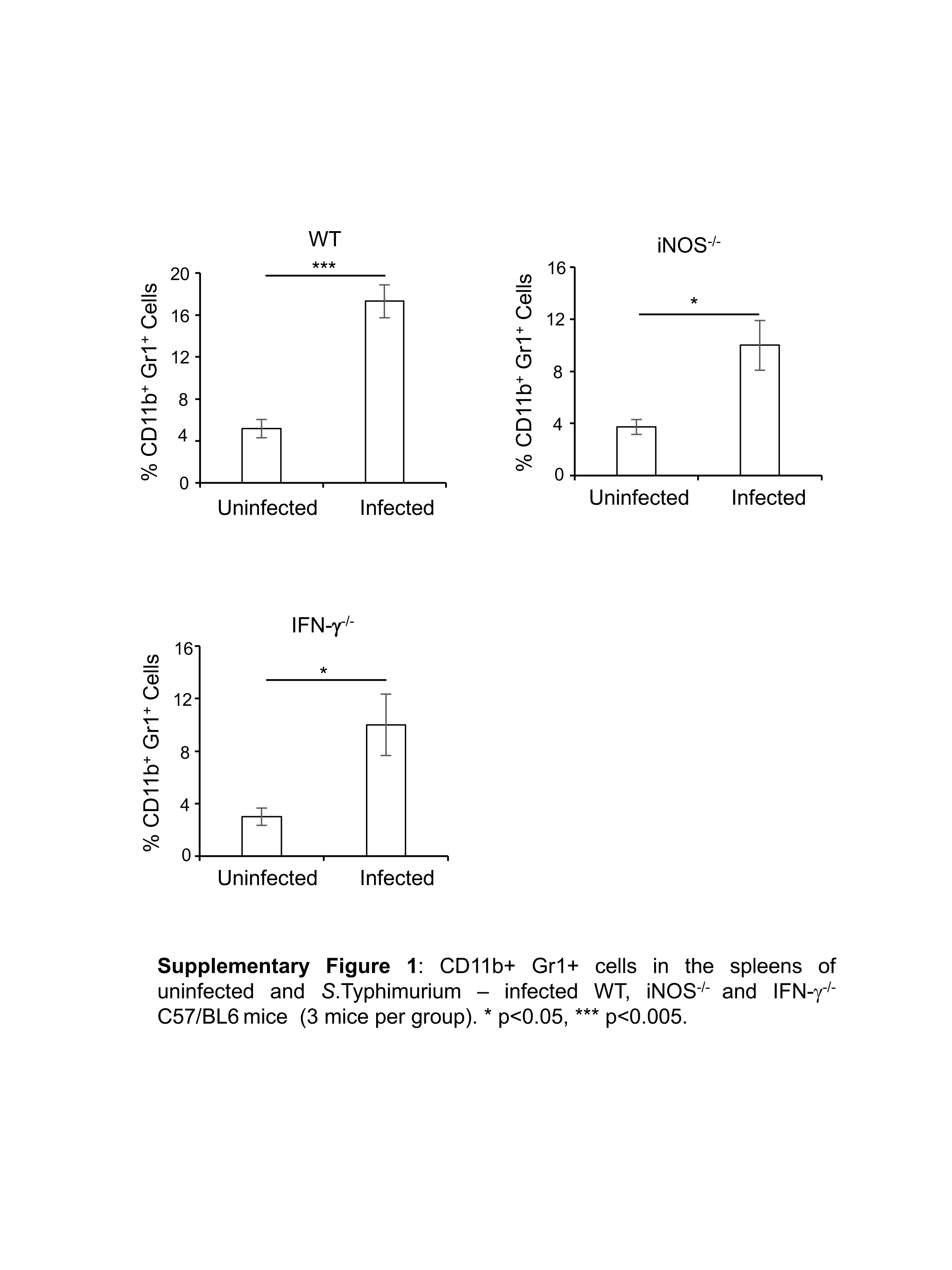

Supplement: Supplementary file 1 [file Image_1.TIF]

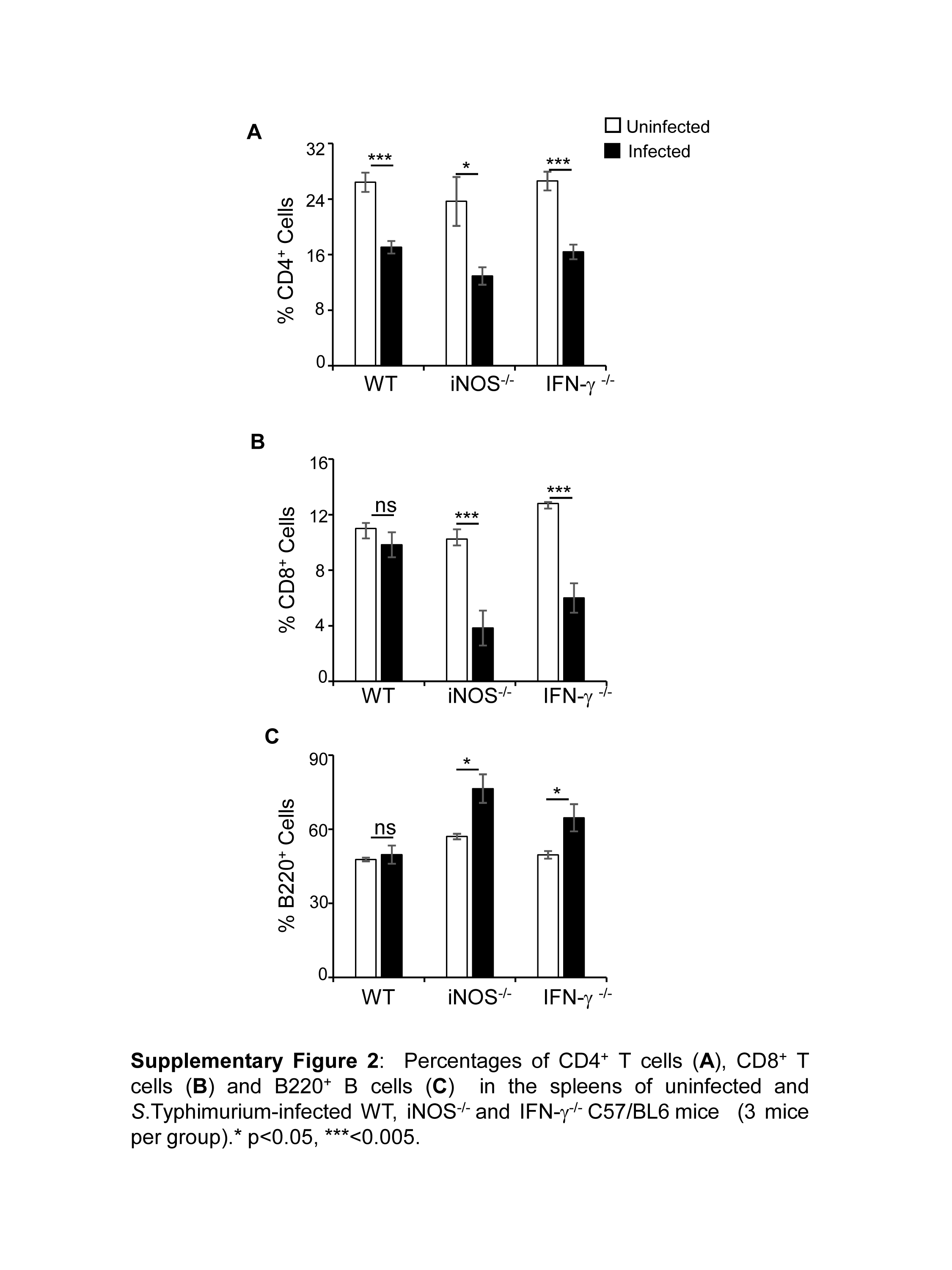

Supplement: Supplementary file 2 [file Image_2.TIF]

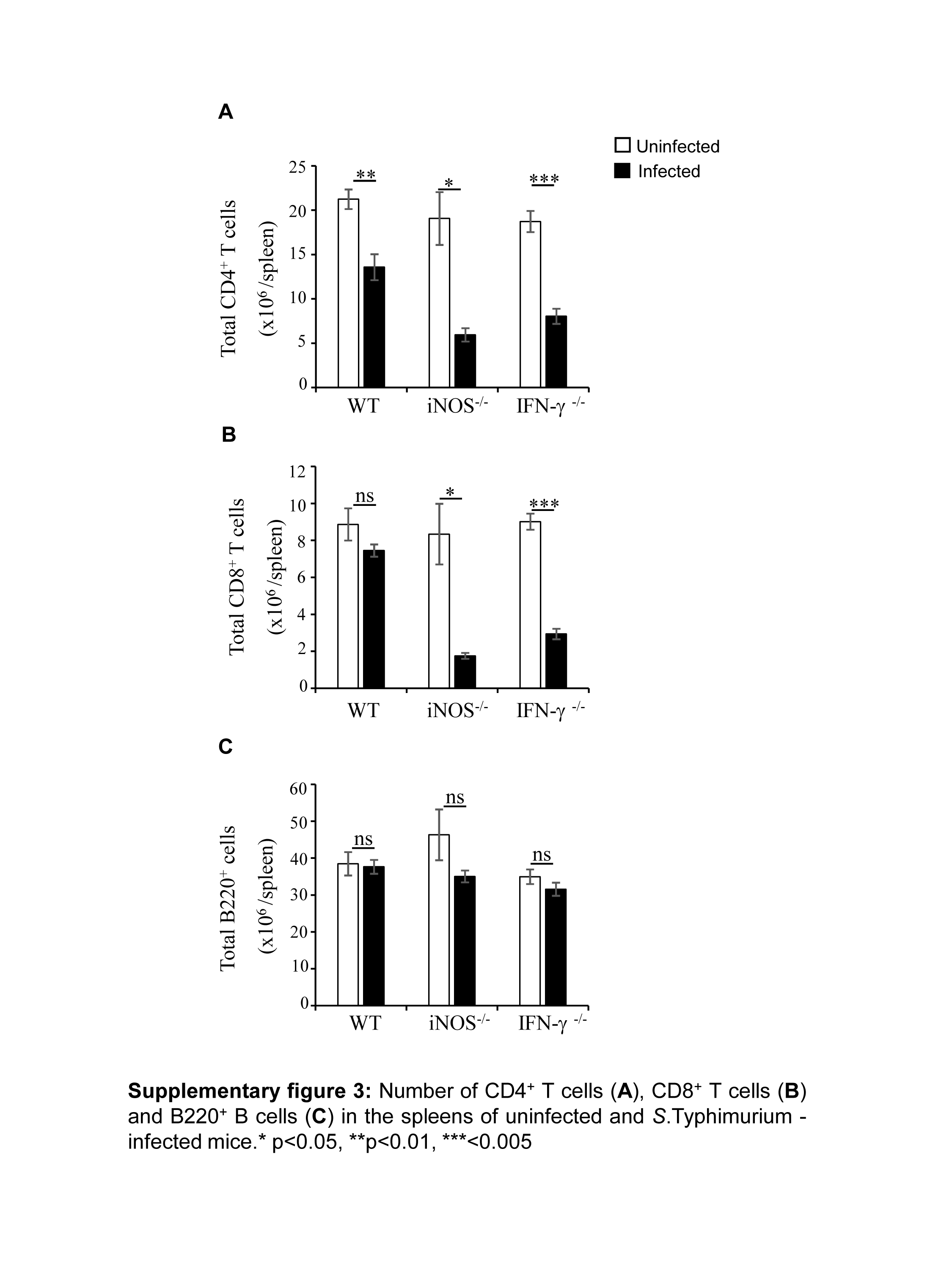

Supplement: Supplementary file 3 [file Image_3.TIF]
